# Association of ADRD Biomarkers and Vessel Skeleton Density (VSD) Derived from Optical Coherence Tomography Angiography (OCTA)

**DOI:** 10.1002/alz70856_107119

**Published:** 2026-01-08

**Authors:** Gabriel A. Vela, Ayantika Banerjee, Monica Goss, Saptaparni Ghosh, Shu Jie Ting, Timothy Kowalczyk, Jared M Zucker, Ana Collazo Martinez, Claudia L Satizabal, Alexa S Beiser, Amir H Kashani, Sudha Seshadri

**Affiliations:** ^1^ Glenn Biggs Institute for Alzheimer's & Neurodegenerative Diseases, University of Texas Health Science Center, San Antonio, TX, USA; ^2^ Framingham Heart Study, Framingham, MA, USA; ^3^ Glenn Biggs Institute for Alzheimer's & Neurodegenerative Diseases, University of Texas Health Science Center, San Antonio, TX, USA; ^4^ University of Texas Health San Antonio, San Antonio, TX, USA; ^5^ The Framingham Heart Study, Framingham, MA, USA; ^6^ Boston University Chobanian & Avedisian School of Medicine, Boston, MA, USA; ^7^ Johns Hopkins University, Baltimore, MD, USA; ^8^ Glenn Biggs Institute for Alzheimer's & Neurodegenerative Diseases, University of Texas Health Science San Antonio, San Antonio, TX, USA; ^9^ Department of Biostatistics, Boston University School of Public Health, Boston, MA, USA; ^10^ Department of Neurology, Boston University Chobanian & Avedisian School of Medicine, Boston, MA, USA; ^11^ Johns Hopkins, Baltimore, MD, USA; ^12^ Department of Neurology, Boston University School of Medicine, Boston, MA, USA; ^13^ Framingham Heart Study, Framingham, Boston, MA, USA; ^14^ Department of Neurology, University of Texas Health Sciences Center, San Antonio, TX, USA, San Antonio, TX, USA

## Abstract

**Background:**

Vessel skeletal density (VSD), derived from Optical Coherence Tomography Angiography (OCTA), reflects capillary density and has been linked to cognitive performance and cerebrovascular health. VSD is considered a potential early biomarker for small vessel disease (SVD), detecting microvascular changes before traditional imaging. Blood‐based biomarkers, such as neurofilament light chain (NfL), glial fibrillary acidic protein (GFAP), and phosphorylated tau (pTau181), are markers of neuronal injury, tau pathology, and neuroinflammation, and have been shown to be elevated in neurodegenerative diseases, including Alzheimer's disease and SVD. However, it is unclear whether neurodegeneration, neuroinflammation, and AD pathology contribute to changes in the retinal microvasculature. This study investigated the association between NfL, pTau181, and GFAP with OCTA‐derived VSD in older adults from the Framingham Heart Study (FHS).

**Methods:**

Participants from the FHS Offspring cohort exam cycle 10 (*n* = 718, mean age: 65.7 ± 6.7 years) underwent OCTA imaging to assess VSD. Blood samples were collected at exam cycle 9 and analyzed for NfL, pTau181, and GFAP levels using Quanterix Simoa assays. To assess the association between VSD and blood‐based biomarkers, we performed a series of multivariate linear regression models. Model 1 was adjusted for age, sex, time between OCTA and exam 9, and eGFR (for NfL only), while Model 2 additionally adjusted for smoking, hypertension, and diabetes mellitus. Statistical significance was set at a 5% level.

**Results:**

NfL was not significantly associated with VSD (Model 1: (β ± standard error: ‐0.001 ± 0.001, *p* =  0.170; Model 2: ‐0.001 ± 0.001, *p* =  0.186). We observed a significant interaction between NfL and age (*p* = 0.045). In participants younger than 66 years only, those in the highest as compared to the lowest NfL quartile had significantly lower VSD (‐0.0036 ± 0.0014, *p* =  0.010). In contrast, no significant association was observed in participants aged 66 years and older. Our study found no significant association between either GFAP or pTau181 and VSD.

**Conclusion:**

These findings suggest that higher NfL levels may be associated with reduced retinal capillary density in younger individuals and may indicate a link between neurodegeneration and microvascular health.

